# A Lipid-Based Droplet Processor for Parallel Chemical Signals

**DOI:** 10.1021/acsnano.1c08217

**Published:** 2021-11-17

**Authors:** Idil Cazimoglu, Michael J. Booth, Hagan Bayley

**Affiliations:** Chemistry Research Laboratory, University of Oxford, Oxford, OX1 3TA, U.K.

**Keywords:** alpha hemolysin, nanopores, synthetic biology, sensors, drug delivery, multisomes, droplet interface bilayers (DIBs)

## Abstract

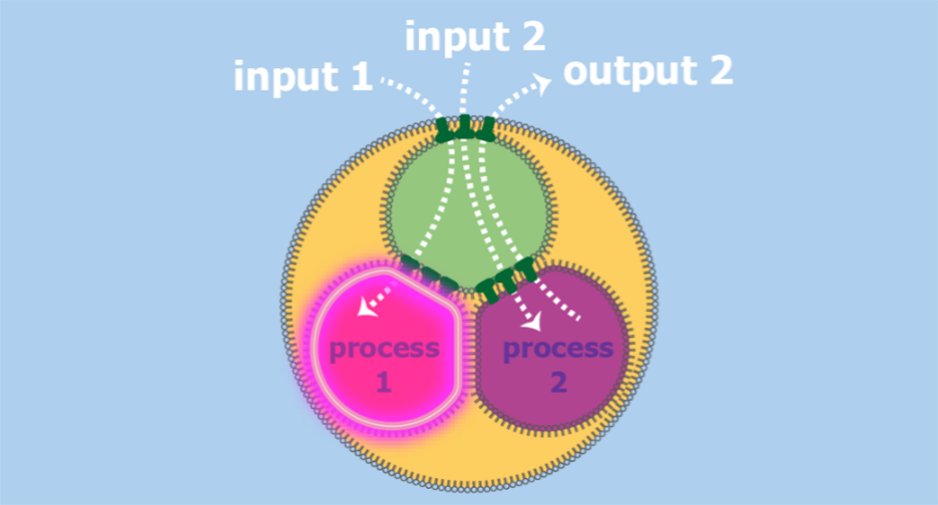

A key goal of bottom-up synthetic biology is to construct cell- and tissue-like structures. Underpinning cellular life is the ability to process several external chemical signals, often in parallel. Until now, cell- and tissue-like structures have been constructed with no more than one signaling pathway. Many pathways rely on signal transport across membranes using protein nanopores. However, such systems currently suffer from the slow transport of molecules. We have optimized the application of these nanopores to permit fast molecular transport, which has allowed us to construct a processor for parallel chemical signals from the bottom up in a modular fashion. The processor comprises three aqueous droplet compartments connected by lipid bilayers and operates in an aqueous environment. It can receive two chemical signals from the external environment, process them orthogonally, and then produce a distinct output for each signal. It is suitable for both sensing and enzymatic processing of environmental signals, with fluorescence and molecular outputs. In the future, such processors could serve as smart drug delivery vehicles or as modules within synthetic tissues to control their behavior in response to external chemical signals.

Lipid-bounded aqueous compartments have applications in drug delivery as well as bottom-up synthetic biology. They can be interfaced to an external aqueous environment through a lipid bilayer and may contain subcompartments.^1^ Those devised for drug delivery are designed to release their contents, which can be passive through biodegradation^2^ or targeted through structural degradation coupled to environmental triggers such as pH, ultrasound, or other external signals.^2−5^ Lipid-based compartments have also been developed to exhibit cell-like functions.^6,7^ For example, they can employ nanopore-forming membrane proteins to release contents without structural degradation in response to a trigger.^1,5^ They may also receive signals from the environment to activate internal chemical processes such as ATP generation,^8−11^ protein expression through transcription and translation,^11,12^ or glucose metabolism.^13^

Multicompartment structures might execute more complex functions, by acting as synthetic tissues.^14^ Additional compartments not only can carry out an increased number of individual functions but also can collectively exhibit emergent properties.^15,16^ Most of the work on multicompartment structures has been conducted using bilayer-connected droplets in an external lipid-containing oil.^17^ Networks of droplets interconnected by these droplet interface bilayers (DIBs) can be generated manually^18,19^ or by 3D-printing.^16,20^ Within oil, these structures can respond to external light,^19,21^ or mechanical^22^ or electrical^15^ stimuli, but to handle inputs from water-soluble signaling molecules, multicompartment structures must operate in an aqueous environment. Accordingly, multicompartment structures consisting of aqueous droplet compartments inside a lipid-containing oil drop have been generated in water.^23−25^ The compartments are connected to each other and the external environment through lipid bilayers. Multicompartment structures have been formed that degrade by design in response to an external pH or temperature change.^23^ They can also receive^23^ or send^26^ chemical signals from and to the external environment through size-selective nanopores formed by alpha-hemolysin (αHL) in their lipid bilayers.

Current compartmented systems, including multicompartment structures,^27^ have at most a single signaling pathway and carry out a single task. By contrast, natural cells and tissues receive, process, and produce several chemical signals, often simultaneously, with little or no cross-talk.^28−30^ A major limitation to the development of synthetic structures that can process multiple external signals is the slow diffusion of molecular signals across lipid bilayer membranes, using embedded protein nanopores.^26,31,32^ Here, we have optimized the application of protein nanopores to allow the rapid movement of molecular signals across several lipid bilayers. This has allowed us to produce multicompartment droplet processors for parallel chemical signals, with dedicated signal transmission and processing compartments (Figure 1a,b).

**Figure 1:**
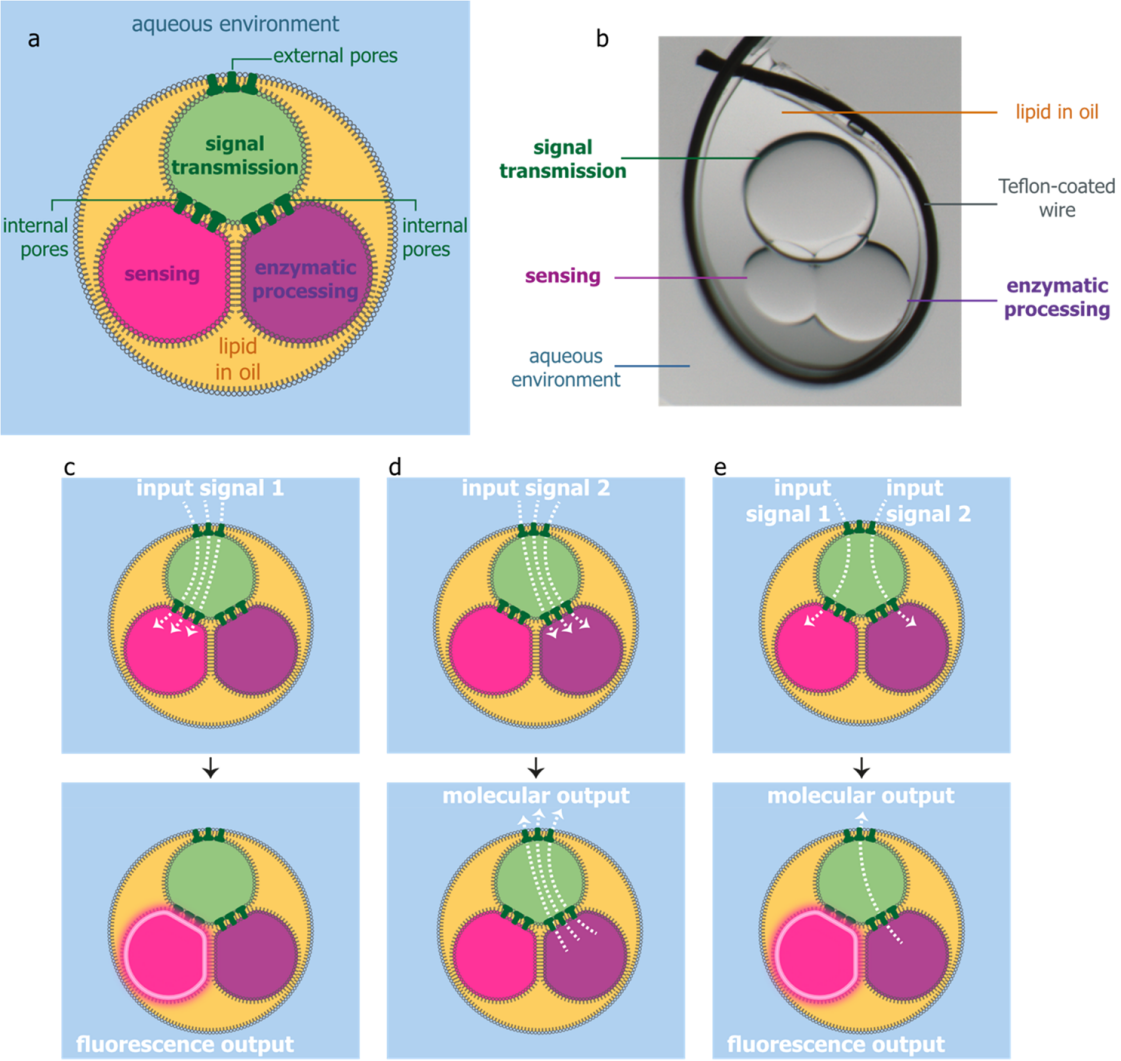
Three-compartment droplet processor for chemical signals, containing sensing and enzymatic processing compartments. (a) A signal transmission compartment enables fast communication between the processing compartments and the external environment *via* α-hemolysin (αHL) nanopores. (b) Top view of a three-compartment droplet processor contained in an oil drop suspended on a Teflon-coated silver wire loop within an aqueous environment. Wire diameter = 76 μm. (c) Sensing in a three-compartment droplet processor by intake of chemical input signal 1 and production of a fluorescence output in the sensing compartment. (d) Enzymatic processing in a three-compartment droplet processor by intake of chemical input signal 2 and production of a molecular output and its release into the external environment by the enzymatic processing compartment. (e) Simultaneous sensing and enzymatic processing in a three-compartment droplet processor by intake of both chemical input signals and production of two distinct outputs.

Our processors consist of a droplet network in external water (Figure 1), with three aqueous droplet compartments inside an oil drop, forming bilayers between each other (DIBs) and the external aqueous environment. The signal transmission compartment contains recombinantly expressed αHL, which forms size-selective nanopores connecting this compartment to the external environment and the two processing compartments. The external nanopores allow the exchange of chemicals between the environment and the signal transmission compartment, whereas the internal nanopores allow the exchange of chemicals between the signal transmission compartment and the processing compartments. Chemical input signals 1 and/or 2 introduced from the external aqueous environment diffuse through the external and internal nanopores and reach both processing compartments (Figure 1c–e). Input signal 1 produces a fluorescence output in the sensing compartment (Figure 1c). Input signal 2 is converted by the enzymatic processing compartment, and the molecular output diffuses through the internal and external nanopores into the external environment (Figure 1d). When both input signals are introduced together, both outputs are produced simultaneously (Figure 1e). Fast diffusion of molecular signals through tissue-like structures is a crucial step on the way to their application as drug delivery devices and synthetic tissues.

## Results and Discussion

### Fast Molecular Diffusion through Nanopores between Compartments

The most crucial requirement for our chemical signal processors is effective signal transmission. Each chemical input signal must diffuse through two bilayers to reach a processing compartment. After enzymatic processing, the molecular output must then diffuse through two bilayers again to reach the external environment (a total of four bilayers), where it becomes diluted by ∼18 000-fold before detection. These factors make fast molecular diffusion essential, which in turn requires efficient insertion of nanopores into the bilayers.

Diffusion through αHL nanopores has been shown with Ca^2+^ ions^1,23,33^ and a range of small molecules.^26,31,32,34−36^ In these cases, the nanopores were produced after cell-free expression of αHL monomers, by using heptamers from *Staphylococcus aureus* purified by a lengthy procedure, or by using up to 50–60 μg mL^–1^ of commercially sourced monomers from *S. aureus*. Incomplete diffusion across lipid bilayers was observed in tens of minutes to hours or days. To make our droplet processors feasible, we required much faster diffusion rates.

**Figure 2:**
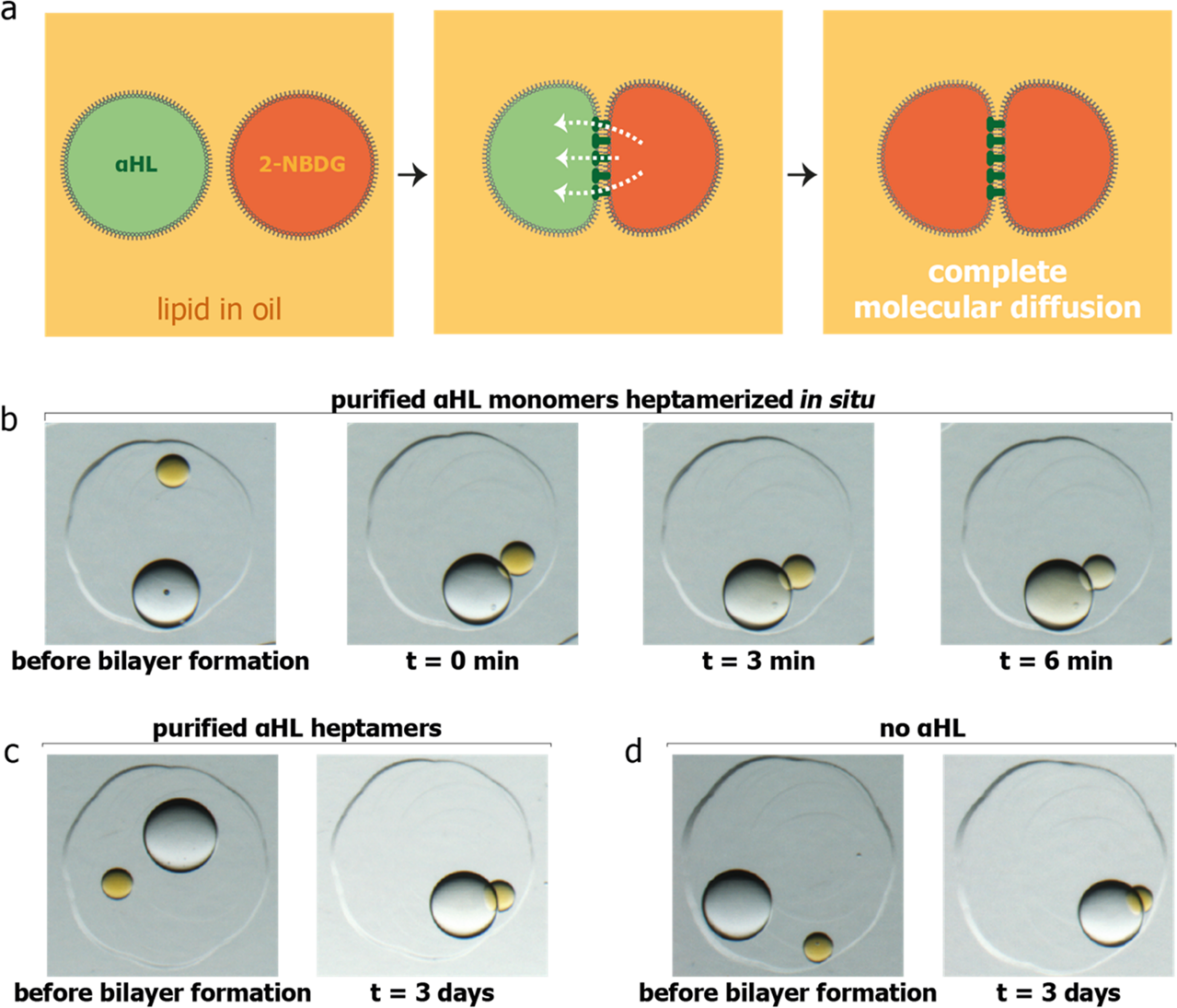
Fast diffusion of molecular signals across lipid bilayers. (a) Bilayer formation between a signal transmission and a signal release compartment within a lipid–oil external environment, leading to diffusion of 2-NBDG molecules through α-hemolysin (αHL) nanopores. (b–d) Bright-field microscopy images of molecular diffusion from compartments containing 2-NBDG into signal transmission compartments containing purified αHL monomers (b), purified αHL heptamers (c), and no αHL (d).

We expressed recombinant αHL in *Escherichia coli* and separated the monomers and heptamers by size exclusion chromatography. We studied diffusion of 2-(*N*-(7-nitrobenz-2-oxa-1,3-diazol-4-yl)amino)-2-deoxyglucose (2-NBDG) molecules through αHL nanopores in droplet interface bilayers within an oil external environment containing 1,2-diphytanoyl-*sn*-glycero-3-phosphocholine (DPhPC) (Figure 2a). Mimicking the dimensions of the compartments within the chemical signal processors we were aiming to build, we formed 8–14 nL (250–300 μm in diameter) signal release compartments containing 1 mM 2-NBDG and 65–144 nL (500–650 μm in diameter) signal transmission compartments containing 200 μg mL^–1^ αHL monomers (Figure 2b) or 200 μg mL^–1^ αHL heptamers (Figure 2c) or no αHL (Figure 2d). Using purified αHL monomers (Figure 2b), we observed molecular diffusion much faster than previously achieved, with complete equilibration of 2-NBDG molecules within 6 min (*n* = 5). We also observed that 2-NBDG diffusion began immediately upon contact of the two compartments and proceeded simultaneously with bilayer formation, as indicated by the increasing contact angle between the compartments^20^ (Supporting Figure 1, Supporting Video 1, Supporting Notes). With αHL heptamers (*n* = 3, Figure 2c) or no αHL (*n* = 3, Figure 2d), transfer of 2-NBDG was not visible after 3 days. The high concentration of αHL, 200 μg mL^–1^, inside the compartments had no adverse effects on the structures or those shown later in this work.

### Exchange of Chemical Signals with the External Environment

**Figure 3:**
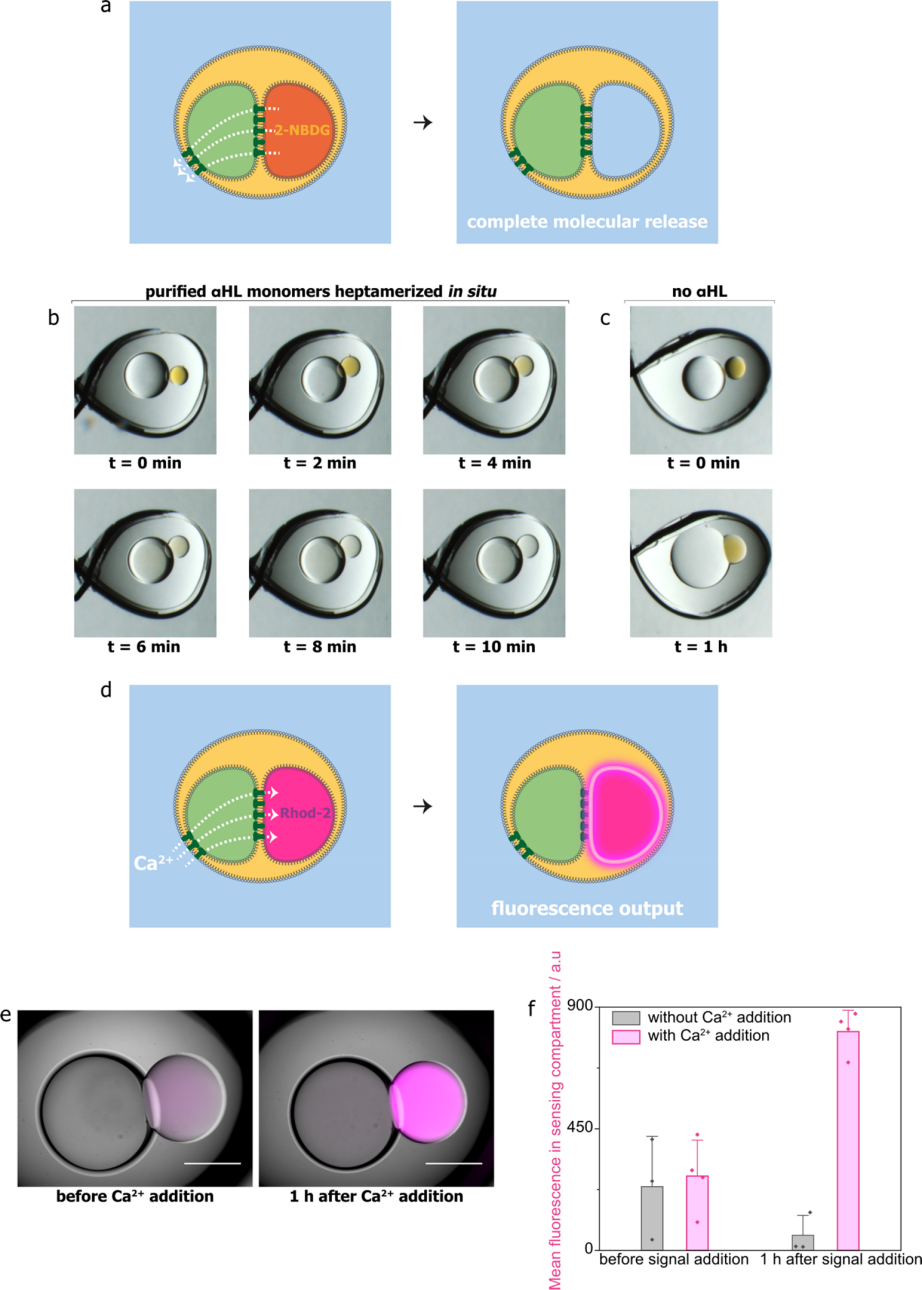
Output and intake of chemical signals in an external aqueous environment. (a) Output of chemical signal 2-NBDG through a signal transmission compartment, leading to its release into the external aqueous environment. (b, c) Bright-field microscopy images of 2-NBDG release into the external aqueous environment through signal transmission compartments with purified αHL monomers (b) and without αHL (c). Wire diameter = 76 μm. (d) Intake of chemical signal Ca^2+^ through the signal transmission compartment, leading to a fluorescence output in the sensing compartment, which contains dextran-conjugated Rhod-2. (e) Composite bright-field and epifluorescence images of a two-compartment processor sensing Ca^2+^ from the external aqueous environment before and 1 h after Ca^2+^ addition. Scale bars = 300 μm. (f) Mean fluorescence values of the sensing compartment before and 1 h after Ca^2+^ addition without (*n* = 3) and with (*n* = 4) Ca^2+^ addition. Error bars represent the standard deviation.

After establishing internal signal transmission between compartments in a lipid–oil external environment, we constructed structures within an aqueous environment. Any chemical output generated by these structures must diffuse through nanopores in two lipid bilayers, first into the signal transmission compartment and then into the external aqueous environment. To mimic this process, we built two-compartment structures with an 8–14 nL (250–300 μm in diameter) signal release compartment containing 2-NBDG and a 65–144 nL (500–650 μm in diameter) signal transmission compartment containing 200 μg mL^–1^ purified αHL monomers (Figure 3a) or no αHL. Complete release of 1 mM 2-NBDG through nanopores in two bilayers to the external environment was observed within 10 min (*n* = 3, Figure 3b, Supporting Video 2, Supporting Notes). Without αHL monomers in the signal transmission compartment, 2-NBDG remained in its original compartment (*n* = 3, Figure 3c).

Our processor would also need to receive external signals through nanopores in two bilayers, first into the signal transmission compartment, then into the sensing compartment to produce a fluorescence output (Figure 3d). To show signal intake and sensing, we built two-compartment structures with a signal transmission compartment containing 200 μg mL^–1^ αHL monomers and a sensing compartment containing 20 μM dextran-conjugated Ca^2+^ indicator Rhod-2 (∼11 000 Da). To minimize reagent use, we included a smaller (14–22 nL in volume, 300–350 μm in diameter) sensing compartment, compared to the larger (65–144 nL in volume, 500–650 μm in diameter) signal transmission compartment. Fluorescence of the Rhod-2 in the sensing compartment increased 3-fold 1 h after 10 mM Ca^2+^ addition (Figure 3e,f) (*n* = 4). When no Ca^2+^ input signal was added, the fluorescence in the sensing compartment did not increase (*n* = 3, Figure 3f). In fact, in this negative control, the fluorescence decreased as a result of excess chelator in the external solution, initially added to prevent the binding of trace metal ions to Rhod-2, diffusing into the sensing compartment (see Supporting Notes).

### Input-Activated Enzymatic Reaction with Fluorescence Output

**Figure 4:**
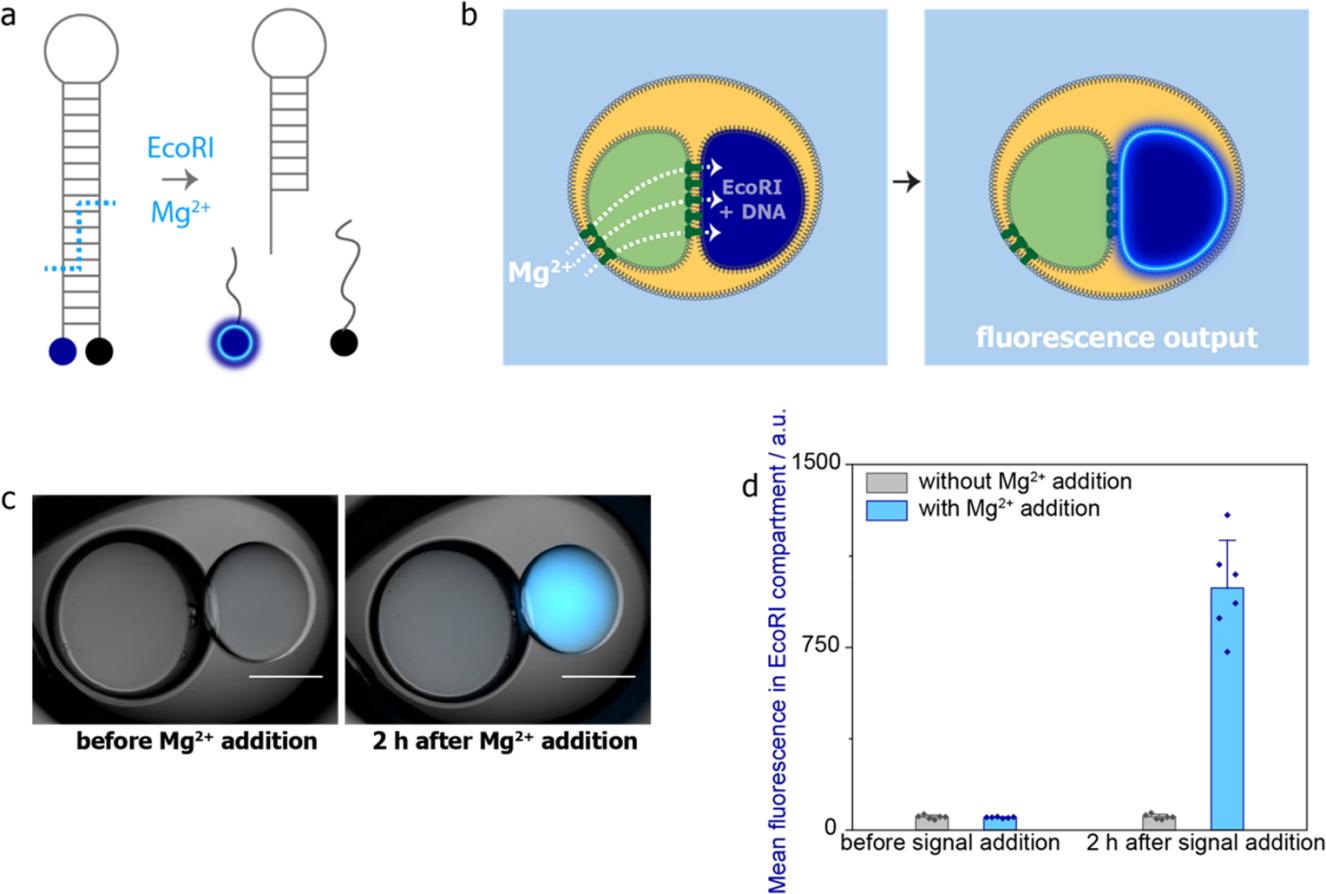
Enzymatic processing by *Eco*RI in two-compartment chemical signal processors: signal intake, signal processing, and fluorescence output. (a) Design of a Mg^2+^-dependent enzymatic process with a fluorescence output. Upon addition of the input signal, a molecular beacon is cleaved by the endonuclease *Eco*RI, leading to fluorophore/quencher pair separation and a fluorescence output signal. (b) Intake of ionic signal Mg^2+^ from the external aqueous environment into a two-compartment processor and activation of enzymatic processing by *Eco*RI to produce a fluorescence output. (c) Composite bright-field and epifluorescence images of a two-compartment chemical signal processor containing *Eco*RI and the DNA substrate before and after Mg^2+^ addition into the external aqueous environment and 2 h at 37 °C. Scale bars = 300 μm. (d) Mean fluorescence values of the *Eco*RI-containing compartment before and 2 h after Mg^2+^ addition and incubation at 37 °C without (*n* = 6) and with (*n* = 6) Mg^2+^ addition. Error bars represent the standard deviation.

The next step was to couple signal intake with activation of an enzymatic process. We chose a restriction endonuclease, *Eco*RI, which requires Mg^2+^ as a cofactor^37^ and had not been encapsulated in synthetic biological systems before. As a substrate, we designed a molecular beacon based on a previously published sequence:^38^ a DNA hairpin with a fluorophore attached on the 5′ end and a quencher on the 3′ end, containing an *Eco*RI cleavage site 4 and 8 bases away from the 5′ and 3′ ends, respectively.^38^ To produce high quenching efficiency,^39^ we used the fluorophore cyanine 5 and the quencher BHQ-3.^40^ Upon cleavage at the *Eco*RI site, the fluorophore/quencher pair separates due to DNA denaturation, resulting in a fluorescence signal (Figure 4a).

We built two-compartment chemical signal processors with a signal transmission compartment containing αHL monomers and an enzymatic processing compartment containing 400 U mL^–1^*Eco*RI and 400 nM DNA substrate. To minimize reagent use, we included 14–22 nL (300–350 μm diameter) enzymatic processing compartments and 65–144 nL (500–650 μm diameter) signal transmission compartments containing 200 μg mL^–1^ αHL monomers. Mg^2+^ input signal added to the external aqueous environment diffused through the signal transmission compartment into the processing compartment, where it induced the DNA cleavage reaction and the fluorescence output (Figure 4b). Fluorescence in the *Eco*RI compartment increased 19-fold 2 h after 10 mM Mg^2+^ addition (Figure 4c,d) and incubation at 37 °C (*n* = 6). When no Mg^2+^ was added, the fluorescence in the *Eco*RI compartment remained constant (*n* = 6, Figure 4d). Notably, we obtained stable structures despite the use of 100 μg mL^–1^ bovine serum albumin (BSA) in the enzymatic processing compartment. For these structures, we initially used 200 μg mL^–1^ αHL monomers in the signal transmission compartment and did not observe an increase in fluorescence in the *Eco*RI compartment after the addition of external Mg^2+^. We made new structures with a reduced αHL concentration (∼100 μg mL^–1^, see Methods) and were then able to detect the fluorescence signal (Figure 4c,d). This indicated that the fluorescent product was diffusing out of the reaction compartment through the nanopores, which we confirmed by fluorescence measurements on the signal transmission compartment (Supporting Figure 2).

### Input-Activated Enzymatic Reaction with Molecular Output

**Figure 5:**
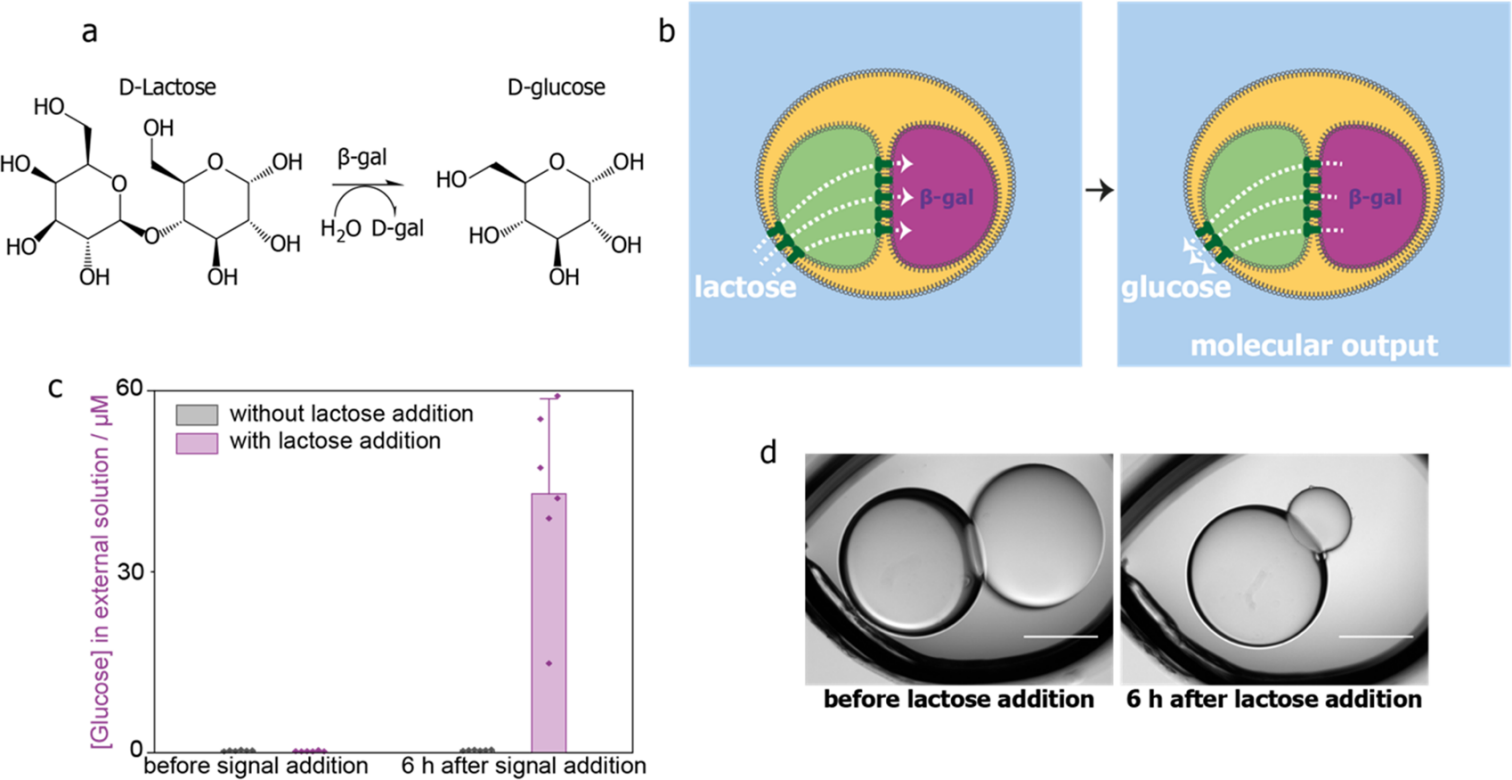
Enzymatic processing by β-galactosidase in two-compartment processors: signal intake, signal processing, small-molecule production, and release. (a) Hydrolysis of d-lactose by β-galactosidase (β-gal) produces d-galactose (d-gal) and d-glucose. (b) Intake of the molecular input signal lactose from the external environment into a two-compartment processor and its enzymatic processing and release of the product glucose into the external environment. (c) Concentration of glucose in the external aqueous environment before and 6 h after lactose addition and incubation at 37 °C, without (*n* = 6) and with (*n* = 6) lactose addition. Error bars represent the standard deviation. (d) Bright-field microscopy images of a two-compartment processor containing β-galactosidase before and after lactose addition and processing. Scale bars = 300 μm.

Having shown ionic signal intake and enzymatic activation in two-compartment processors, we next aimed to demonstrate molecular signal intake, enzymatic turnover, and output of the product molecule into the external environment. We built two-compartment processors with a signal transmission compartment containing 200 μg mL^–1^ αHL monomers and an enzymatic processing compartment containing 700 μg mL^–1^ (30 U mL^–1^) β-galactosidase, which hydrolyzes lactose into glucose and galactose (Figure 5a). The lactose input signal, added to the external aqueous environment, diffused through nanopores in two bilayers, first to the signal transmission compartment and then to the processing compartment, where it was hydrolyzed. The product glucose was then released back through the same two bilayers into the external environment as a molecular output (Figure 5b). In our setup, the external environment was 800 μL in volume. As the product glucose would be diluted upon release to the external environment, we included larger enzymatic processing compartments (34–54 nL in volume, 400–470 μm in diameter) than those we previously used for fluorescence output. Using these larger β-galactosidase compartments, the product glucose was still diluted by ∼18 000-fold upon release. We used signal transmission compartments of the same size as previously used. After 40 mM lactose addition and incubation at 37 °C for 6 h, glucose was detected in the external environment by using a commercial assay (*n* = 6, Figure 5c). When no lactose was added, no glucose was detected (*n* = 6, Figure 5c). In processors incubated with lactose, the β-galactosidase compartment shrank (Figure 5d). We attribute this observation to the increased osmotic pressure of the external aqueous environment. On the other hand, the signal transmission compartment, interfaced directly with the external environment through αHL nanopores, was better able to take in solutes and reach osmotic equilibrium. The processors remained intact despite the volume changes.

### Orthogonal Processing of Two Input Signals

**Figure 6:**
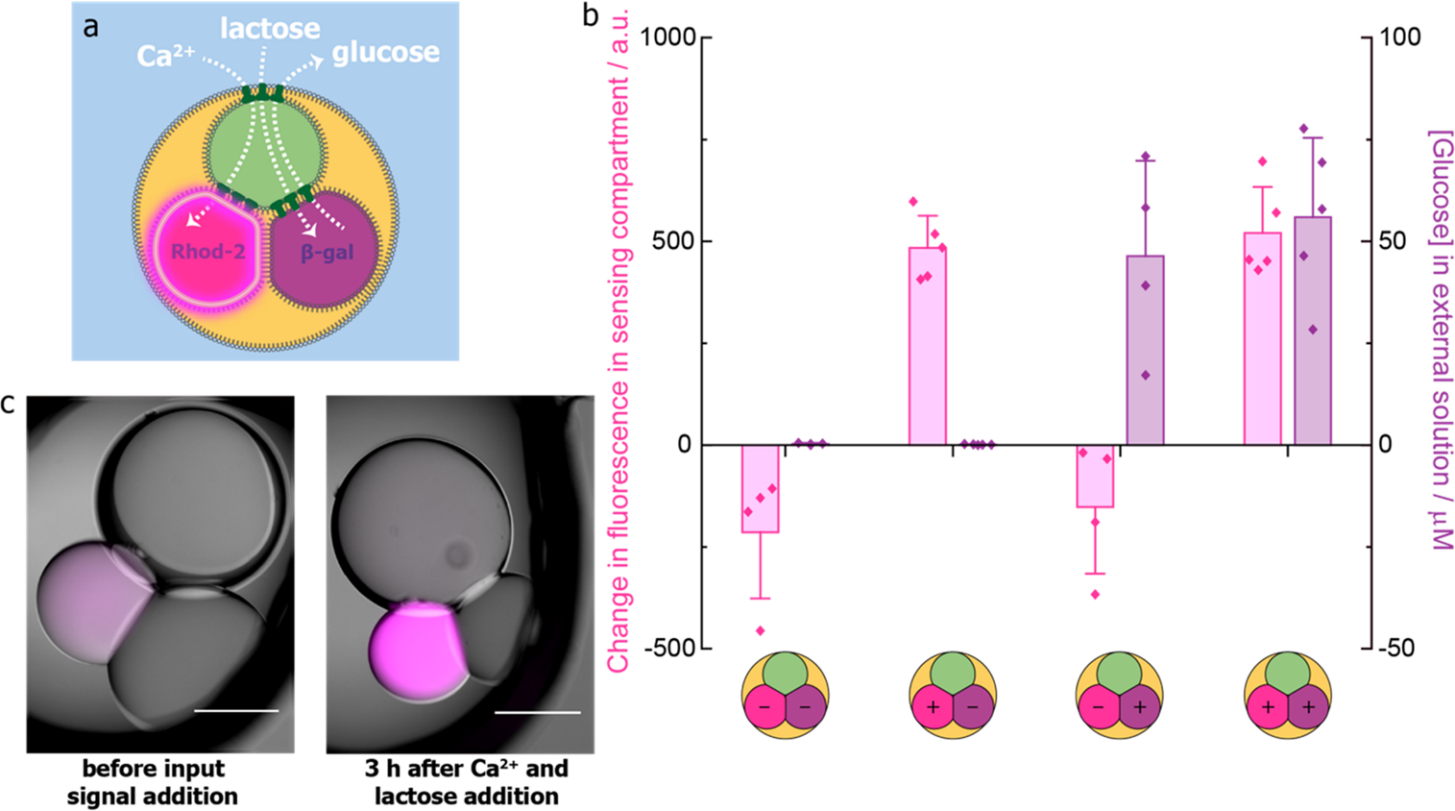
Independent and simultaneous sensing and enzymatic processing in three-compartment processors. (a) Three-compartment processor containing signal transmission, sensing, and enzymatic processing compartments. The intake of Ca^2+^ leads to a fluorescence output in the sensing compartment containing Rhod-2, whereas the intake of lactose leads to the production of glucose by β-galactosidase and its release into the external environment. (b) Changes in the mean fluorescence of Rhod-2 compartments and the mean concentration of glucose detected in the external solution after 3 h at 37 °C for three-compartment processors with no input signals (*n* = 4) and with an input of Ca^2+^ only (*n* = 5), lactose only (*n* = 4), and both Ca^2+^ and lactose (*n* = 5). Error bars represent the standard deviation. (c) Composite bright-field and epifluorescence images of a three-compartment processor with a sensing compartment containing Rhod-2 and an enzymatic processing compartment containing β-galactosidase before and after simultaneous addition of Ca^2+^ and lactose input signals and 3 h at 37 °C. Scale bars = 300 μm.

At this stage, we had all the parts required to build a processor that would independently and simultaneously process two external chemical signals. Combining a signal transmission compartment containing 200 μg mL^–1^ αHL monomers, a sensing compartment containing 20 μM dextran-conjugated Rhod-2, and an enzymatic processing compartment containing 700 μg mL^–1^ β-galactosidase, we constructed three-compartment processors (Figure 6a). We tested these processors with all four possible input signal combinations: no input signals, only Ca^2+^, only lactose, and both Ca^2+^ and lactose (Figure 6b). When no input signals were added, fluorescence in the Rhod-2 compartment decreased and no glucose was detected in the external environment, after 3 h at 37 °C (*n* = 4). Addition of only the Ca^2+^ input signal led to a fluorescence output in the Rhod-2 compartment and no glucose was detected in the external environment (*n* = 5). When only the lactose input signal was added, the fluorescence of the Rhod-2 compartment decreased and glucose was detected in the external environment (*n* = 4). When both input signals were added, fluorescence in the Rhod-2 compartment increased (Figure 6b, c) and glucose was detected in the external environment (*n* = 5, Figure 6b). These results demonstrate the orthogonal processing of two chemical inputs within a lipid-bound multicompartment structure.

### Simultaneous Enzymatic Processing of Two Input Signals

**Figure 7:**
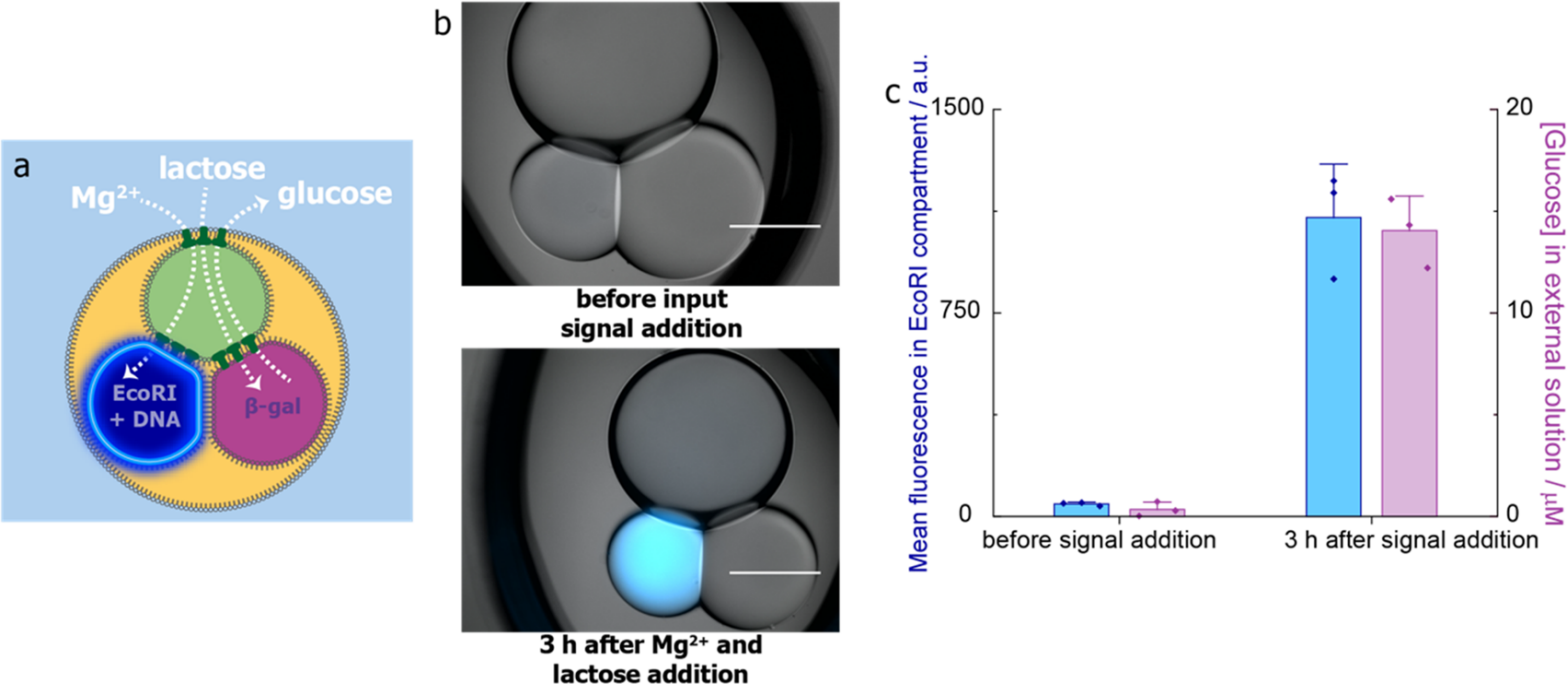
Simultaneous enzymatic processing in three-compartment processors. (a) Three-compartment processor for simultaneous enzymatic processing by *Eco*RI and β-galactosidase activated by input signals Mg^2+^ and lactose, respectively. (b) Composite bright-field and epifluorescence images of a three-compartment processor with *Eco*RI and β-galactosidase processing compartments before and after the simultaneous addition of Mg^2+^ and lactose and 3 h incubation at 37 °C. Scale bars = 300 μm. (c) Mean fluorescence values in the *Eco*RI-containing compartment and the mean concentration of glucose detected in the external solution before and after the simultaneous addition of Mg^2+^ and lactose and 3 h at 37 °C (*n* = 3).

To demonstrate simultaneous enzymatic processing, we constructed processors with a signal transmission compartment containing ∼100 μg mL^–1^ αHL monomers and two enzymatic processing compartments: one containing 400 U mL^–1^*Eco*RI with 400 nM DNA substrate and one containing 700 μg mL^–1^ β-galactosidase (Figure 7a). We had to find a pH value that would allow both reactions to proceed and selected pH 6.5 as a compromise (Supporting Figures 3, 4). The addition of both input signals, 10 mM Mg^2+^ and 40 mM lactose, followed by incubation at 37 °C for 3 h generated increased fluorescence in the *Eco*RI compartment (Figure 7b,c) and glucose in the external environment NBDG, the tetrapotassium salt of BAPTA, and the Amplex(Figure 7c) simultaneously (*n* = 3), despite the suboptimal reaction conditions.

## Conclusion

In summary, we have constructed droplet processors for parallel chemical signals from lipid bilayer-connected compartments from the bottom up, in a modular fashion. By using purified αHL monomers we were able to encapsulate a high concentration of αHL in the signal transmission compartments, achieving rapid movement of ionic and molecular signals across as many as four lipid bilayers. Using our droplet processors, we first demonstrated the release and intake of chemical signals in two-compartment structures. We then encapsulated enzymes within them, and with an ionic input signal, we activated DNA cleavage. We also demonstrated the enzymatic hydrolysis of a molecular input signal and release of the product into the external environment as a molecular output. By combining different components in a modular fashion, we built three-compartment processors that receive and process two different chemical signals in an orthogonal manner, producing two distinct outputs: fluorescence and molecule release. We also showed simultaneous activation of different enzymes in two separate compartments of a three-compartment processor.

The bottom-up synthetic biological system built here, which demonstrates independent and simultaneous processing of more than one signal, serves as a stepping stone in the development of multicompartment systems with complex signal processing capabilities. This work overcomes a major obstacle in bottom-up synthetic biology, by demonstrating the rapid movement of molecular signals across multiple lipid bilayers, facilitated by using recombinant αΗL monomers. The size-selective pores, used at a high concentration, enable fast diffusion of ionic and small-molecule signals, while holding larger molecules such as enzymes within the compartments and ensuring each process takes place in a specific compartment. Therefore, our work also shows that orthogonal processing can be achieved by the appropriate selection of compartment contents, without requiring directional transport of signals. Nonetheless, applications requiring directional diffusion of signaling molecules could be enabled by incorporating additional membrane proteins, such as the mechanosensitive channel of large conductance (MscL),^1,5,41^ and by using other pore-forming techniques, such as the photopolymerization of lipids.^42,43^ For further complexity in signaling, engineered αΗL nanopores might be used at high concentrations to modulate signal transmission with small molecules,^26,44,45^ light,^46^ or other stimuli,^47,48^ enabling future structures to perform more intricate tasks. The processors in this work have been constructed manually and would benefit from high-throughput production methods such as microfluidics^24,25,49^ and 3D-printing.^16,20^ These methods would eliminate the need for assembly by manual manipulation using a Teflon-coated silver wire.

Our processors are modular, robust, and versatile. Lipid-connected droplet compartments in an external aqueous environment often suffer from structural instability,^26,33^ which can be addressed by balancing osmotic pressures during construction^26,35^ but limiting the applicability of these structures in dynamic environments. Our processors incorporate high concentrations of enzymes and membrane proteins, can withstand changing osmotic pressures, and are stable at 37 °C.

Due to their modularity and robustness, the processors might be adapted for a variety of applications. To simultaneously process complex sets of input signals, several processing units could be connected to a large signal transmission compartment (Supporting Figure 5, Supporting Video 3). The platform could be used to build sensors and microreactors. For example, the encapsulation of suitable reporters would allow parallel processing in medical diagnostics, water quality analysis, or the sensing of bacteria. Microreactors could be built by combining compartments that encapsulate multiple independent reactions, sequential reactions forming a cascade, or both. The multicompartment processors might also be incorporated into drug delivery systems or synthetic tissues to enable complex communication with their environment through parallel signal processing. For example, to enhance drug delivery, the processors could be engineered to monitor and integrate multiple biomarkers to produce an output. In the future, these processors could also handle signals between synthetic and living tissues, coordinating them to act as functional hybrid systems.

## Methods

### αHL Expression and Purification

BL21(DE3)pLysS *E. coli* cells (Agilent) were transformed with ∼2 μg of αHL-D8H6 plasmid^50^ without heat shock and incubated on LB-agar plates containing 25 g L^–1^ lysogeny broth (LB) and 15 g L^–1^ agar with antibiotics (50 μg mL^–1^ carbenicillin disodium and 34 μg mL^–1^ chloramphenicol) at 37 °C for 16 h. Single colonies were picked and inoculated into 15 g L^–1^ LB with antibiotics at 37 °C with shaking at 250 rpm until OD ≈ 0.7. The cultures were then induced with 1 mM isopropyl β-d-1-thiogalactopyranoside (IPTG) and incubated at 18 °C with shaking at 250 rpm for 16 h. Between this point and fast protein liquid chromatography (FPLC) purification, all steps were carried out at 4 °C. The cells were pelleted and lysed in 50 mM Tris-HCl, pH 8.0, 0.5 M NaCl, 10 mM imidazole, and 0.1% Triton X-100 followed by the addition of ice-cold MgCl_2_ (final concentration, 5 mM), hen egg white lysozyme (final concentration, 1 mg mL^–1^), and 250 units benzonase.

The resulting lysate was sonicated using an ultrasonic probe with 30 s pulses and 30 s intervals for 3 min followed by centrifugation to separate the supernatant and cell debris. The supernatant was loaded onto a column preloaded with 2 mL of Ni-NTA resin (HisPur) and placed on a rotator disk for 1 h. The column was then washed twice with 15 mL of 50 mM Tris-HCl pH 8.0, 0.5 M NaCl, 10 mM imidazole, and 0.1% Triton X-100. Proteins were eluted in 5 × 1 mL batches with 50 mM Tris-HCl pH 8.0, 0.5 M NaCl, 250 mM imidazole, and 0.1% Triton X-100 and stored at −80 °C until FPLC purification.

To separate monomers and heptamers and exchange the buffer, 500 μL of an elution with high protein concentration, as judged by SDS-PAGE electrophoresis, was loaded at room temperature onto a Superdex 75 100/300 GL (GE Healthcare) gel filtration column, which was equilibrated and run in 10 mM Tris-HCl pH 8.0/200 mM NaCl at a flow rate of 0.5 mL min^–1^ and eluted as 0.5 mL volume per fraction. Monomeric and heptameric αHL were detected by ultraviolet absorption at 254 and 280 nm and by SDS-PAGE electrophoresis. Protein concentrations were measured using a Nanodrop. The fractions with highest concentrations (200–400 μg mL^–1^) were stored as 5–10 μL aliquots in Protein LoBind tubes (Eppendorf) at −80 °C.

### Compositions of Solutions

DPhPC (Avanti Polar Lipids) was stored as a powder at −80 °C. Hexadecane (Merck) and silicone oil AR20 (Wacker) were filtered before use through 0.22 μm poly(ether sulfone) filters (Corning) under vacuum. Lipid-oil solutions were prepared by dissolving the desired amount of lipid in chloroform in isopropanol-cleaned glass vials and evaporating the solvent under a slow stream of nitrogen gas while manually rotating. The films were dried under vacuum overnight and stored under argon in Teflon-capped glass vials (Supelco). For use, a film was dissolved by sonication for 45 min in 65:35 v:v silicone oil/hexadecane to give 2 mM DPhPC (5 mM for Supporting Figure 1).

Rhod-2 dextran conjugate (∼11 000 Da) was from AAT Bioquest. 2-NBDG, the tetrapotassium salt of 1,2-bis(*o*-aminophenoxy)ethane-*N*,*N*,*N*′,*N*′-tetraacetic acid (BAPTA), and the Amplex Red glucose/glucose oxidase assay kit were from Invitrogen. *Eco*RI-HF was from New England Biolabs. All other reagents used in aqueous solutions were purchased from Merck. β-Galactosidase from *A. oryzae* was purified with a PD-10 desalting column (GE Healthcare). The custom-made DNA substrate was purchased from ATDBio. It was designed based on a sequence described previously,^38^ but with the fluorophore cyanine 5 on the 5′ end and the quencher black hole quencher (BHQ-3) on the 3′ end.

An individual aliquot of αHL was thawed on ice for each experiment and diluted with 10 mM Tris-HCl pH 8.0/200 mM NaCl to give 200 μg mL^–1^ αHL. For processors involving a compartment containing *Eco*RI and DNA, the αHL was diluted further to a concentration (∼100 μg mL^–1^) at which the equilibration of 2-NBDG across a bilayer (Figure 2) took 20–30 min.

All other compartments and external aqueous environments contained 50 mM MES/100 mM NaCl at pH 6.5 for experiments with *Eco*RI or 2-NBDG and pH 5.5 for all other experiments. All processing compartments and external environments contained 2 μM BAPTA for experiments using Rhod-2. 2-NBDG was used at 1 mM. Rhod-2 was used at 20 μM. In compartments containing *Eco*RI and DNA, 400 U mL^–1^*Eco*RI, 400 nM DNA substrate, and 100 μg mL^–1^ BSA were used. β-Galactosidase was used at 700 μg mL^–1^. Input signals were at final concentrations of 0 or 10 mM CaCl_2_ (for Rhod-2), 0 or 10 mM MgCl_2_ (for *Eco*RI), and 0 or 40 mM d-lactose (for β-galactosidase) in the same buffer conditions as each external solution into which they were added.

### Formation of Processors and Processor Mimics

Chambers were formed as previously described.^26^ After the addition of external aqueous solution to each chamber, lipid-containing oil drops (∼1.5 μL) were formed on Teflon-coated silver wire loops, zapped with an antistatic gun, and incubated to form a monolayer for at least 5 min. About 0.5 μL of each oil drop was pipetted out to reduce the drop volumes. The oil drops were then incubated for at least another 5 min.

Compartments were formed in the lipid-containing oil inside poly(methyl methacrylate) (PMMA) wells, by using a Gilson P2 pipet. 2-NBDG, β-galactosidase, and Rhod-2 compartments were formed first and incubated in the PMMA wells for at least 10 min. *Eco*RI and αHL compartments were formed later and not incubated. To make each processor, all compartments were simultaneously transferred into the oil drop using a pipet.

For processors with a molecular output, sample solution was pipetted out of each chamber before signal addition for analysis. For all processors, the total external solution after signal addition was 800 μL. The processors were incubated for the specified time, at 37 °C where indicated; then sample solutions were pipetted out of each chamber again for analysis. For all processors in Figures 3e,f, 4, 5, 6, and 7, bright-field and/or epifluorescence microscopy images were taken before and after signal addition and incubation. A minimum incubation time of 30 min was allowed after processor formation to ensure that the structures had reached a stable configuration before imaging.

### Fluorescence Output Detection and Analysis

Images for fluorescence outputs were taken using a Leica DMi8 epi fluorescence microscope. For 2-NBDG, the filter cube GFP was used (ex: 450–490 nm, em: 500–550 nm), for Rhod-2 DSRED was used (ex: 540–552, em: 567–643 nm), and for *Eco*RI+DNA Y5 was used (ex: 590−650, em: 662−738). For quantification, the fluorescence compartments were detected and analyzed on Fiji/ImageJ by a custom script followed by manual verification and, in rare cases involving artifacts caused by an air bubble or the silver wire, correction. The script applied Gaussian blur with a standard deviation of 4 and Moments automatic thresholding to each fluorescence image, then used the built-in particle analysis tool of Fiji/ImageJ to detect and record a region of interest (ROI) for each particle with an area of 20 000–70 000 μm^2^ and a circularity of 0.15–1.00. Each ROI was then applied to its corresponding original fluorescence image to record the average fluorescence value within the compartment area. For two-compartment processors with an *Eco*RI+DNA compartment, an additional ROI was defined by drawing a line across the diameter of each signal transmission compartment. The ROIs were then applied to their corresponding fluorescence images to obtain fluorescence values across each signal transmission compartment.

### Glucose Detection

Glucose detection was performed using the Invitrogen Amplex Red glucose/glucose oxidase assay kit according to the manufacturer’s instructions. For each sample, a calibration curve with a matching buffer was used. For example, samples from a processor that received a lactose input were compared to a glucose calibration curve that also contained lactose. To avoid the nonlinear range of the assay, samples with [glucose] > 30 μM were diluted and remeasured.

### Contact Angle Measurements

Contact angle measurements were obtained as described previously.^20^ Briefly, the contact angle (θ) formed between two compartments was calculated from the radius of each compartment (*R*_1_, *R*_2_) and the center-to-center distance (*L*) by using the formula^51^
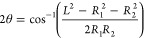


The contact angles were calculated from bright-field microscopy images using a custom-written script in MATLAB (Mathworks), which estimated the radii *R*_1_ and *R*_2_ of the two compartments by circle fitting and the center-to-center distance *L* from the distance between the two fitted circles around the compartments. The script was also used to record the average fluorescence value within the detected compartment area.

### *Eco*RI Assays

Using a 96-well plate, 110 μL reaction mixes were prepared in 91 mM NaCl, 91 μg mL^–1^ BSA, and 45 mM MES pH 5.5 or MES pH 6.5 or Tris pH 8.0, with 0 or 10 mM MgCl_2_. A 364 nM DNA substrate and 364 U mL^–1^*Eco*RI were used. The plate was covered and placed in a Tecan Infinite M1000 Pro microplate reader at 37 °C, and fluorescence measurements were taken from the bottom of the plate every 5 min with ex = 645–655 nm and em = 665–675 nm.

## Supporting Information Available

Supporting figures of molecular diffusion across a droplet interface bilayer, diffusion of product after DNA cleavage by *Eco*RI, *Eco*RI activity, β-galactosidase activity, a multicompartment processor mimic; supporting notes on mathematical considerations of molecular diffusion in various systems containing droplet interface bilayers; descriptions of supporting videos (PDF)
Supporting video demonstrating 2-NBDG diffusion through αHL pores within a lipid–oil external environment (MPG)
Supporting video demonstrating 2-NBDG release through αHL pores in two bilayers within an aqueous external environment (MPG)
Supporting video demonstrating self-assembly of a structure with one large and multiple small compartments within an aqueous external environment (MPG)

## Supplementary Material

nn1c08217_si_001.pdf

nn1c08217_si_002.mpg

nn1c08217_si_003.mpg

nn1c08217_si_004.mpg

## Notes

This work was previously submitted to the preprint server bioRxiv: Cazimoglu, I.; Booth, M. J.; Bayley, H. A Lipid-Based Parallel Processor for Chemical Signals. *bioRxiv* 2021. DOI: 10.1101/2021.05.05.442835 (accessed Oct 4, 2021).
