# Supplementary material for: A Lipid-Based Droplet Processor for Parallel Chemical Signals
Source: ACS Nano. 2021 Nov 17;15(12):20214–24. doi: 10.1021/acsnano.1c08217 (PMC8717631; doi:10.1021/acsnano.1c08217)
Supplement: Supplementary file 1 — nn1c08217_si_001.pdf [file nn1c08217_si_001.pdf]

Supporting Information

A Lipid-Based Droplet Processor for Parallel Chemical Signals

Idil Cazimoglu, Michael J. Booth, Hagan Bayley**

Chemistry Research Laboratory, University of Oxford, Oxford, OX1 3TA, UK

Supporting Figures

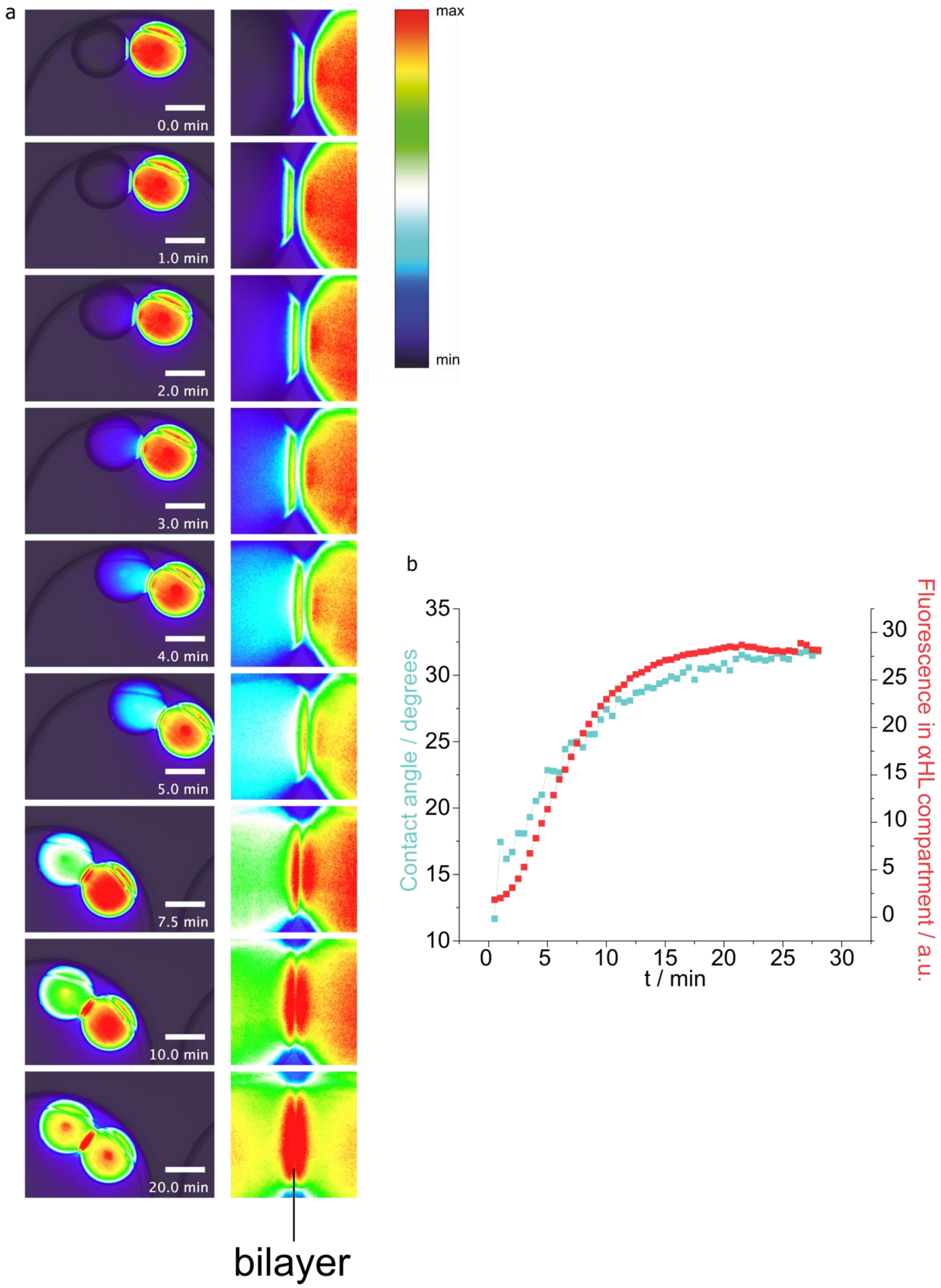

Supporting Figure 1. a Composite bright-field and epifluorescence time-lapse microscopy images of molecular diffusion from a signal release compartment containing 2-NBDG into a signal transmission compartment containing purified α-hemolysin (αHL) monomers, in an

external lipid-oil environment, as seen in Supporting Video 1. Magnified images of bilayers corresponding to each time point are also shown. Molecular diffusion began immediately upon contact of the compartments, before the bilayer had expanded to its final area. A plume of fluorescence was initially observed near the bilayer in the αHL-containing compartment, followed by mixing inside the compartment. Scale bars = 300 µm. **b** Plots of the contact angle between the two compartments and fluorescence in the signal transmission compartment over time. Increasing contact angle indicates increasing bilayer area. Bilayer formation began when the two compartments touched, and proceeded over several minutes. The rate of bilayer formation was high at the beginning and decreased as the bilayer approached its maximum size. The rate of molecular diffusion started low and rapidly increased as more pores formed in the bilayer. After the maximum number of pores had formed, molecular diffusion reached a constant rate. Then, as the concentration gradient of the molecules across the bilayer, ΔC, became smaller, molecular diffusion slowed down.

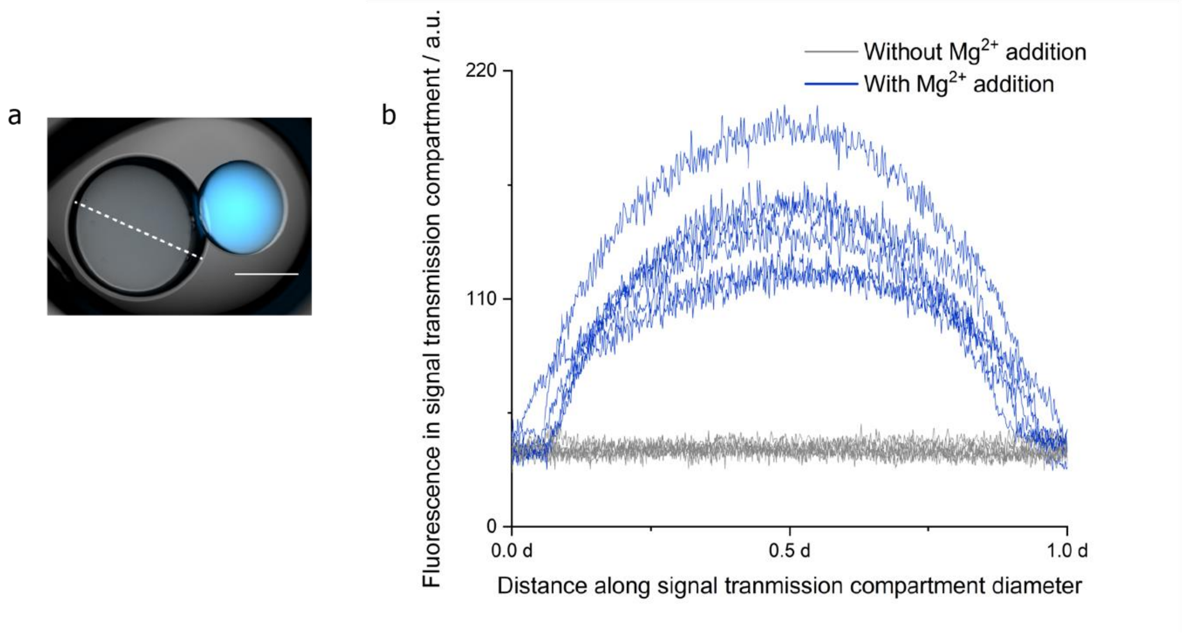

Supporting Figure 2. Analysis of fluorescence in the signal transmission compartment of two-compartment processors containing an *Eco*RI+DNA compartment as seen in Figure 4. **a** Fluorescence of each signal transmission compartment was analyzed by drawing a line across the compartment, indicated as dashed white line. **b** Fluorescence values across signal transmission compartments, d = compartment diameter. In processors given the Mg^{2+} input signal, the fluorescent product from the *Eco*RI reaction was observed in the signal transmission compartment ($n = 6$). No fluorescence was observed in the signal transmission compartment if Mg^{2+} was not added ($n = 6$).

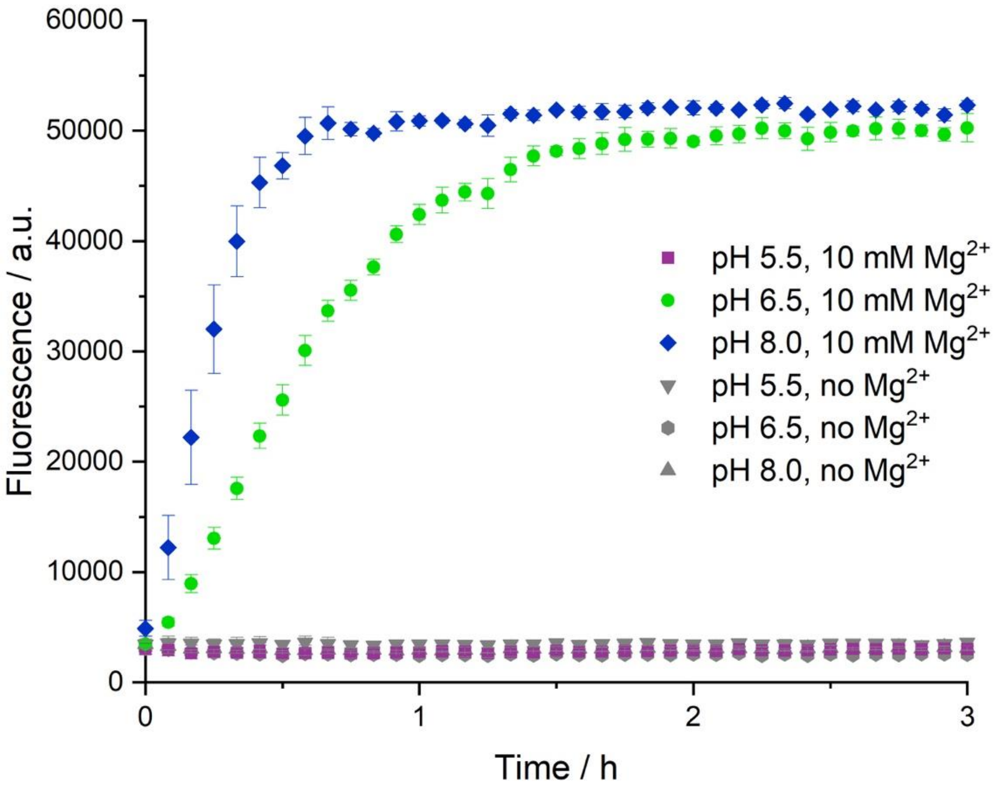

Supporting Figure 3. *Eco*RI activity at various pH values, with or without the co-factor Mg^{2+}. The fluorescent product was measured. DNA cleavage by *Eco*RI did not proceed without Mg^{2+} (n = 3 for each pH condition). The *Eco*RI enzyme was highly active at pH 8.0 (n = 3). Reduced activity was observed at pH 6.5 (n = 3), whereas no activity was observed at pH 5.5 (n = 3). Error bars represent standard deviation.

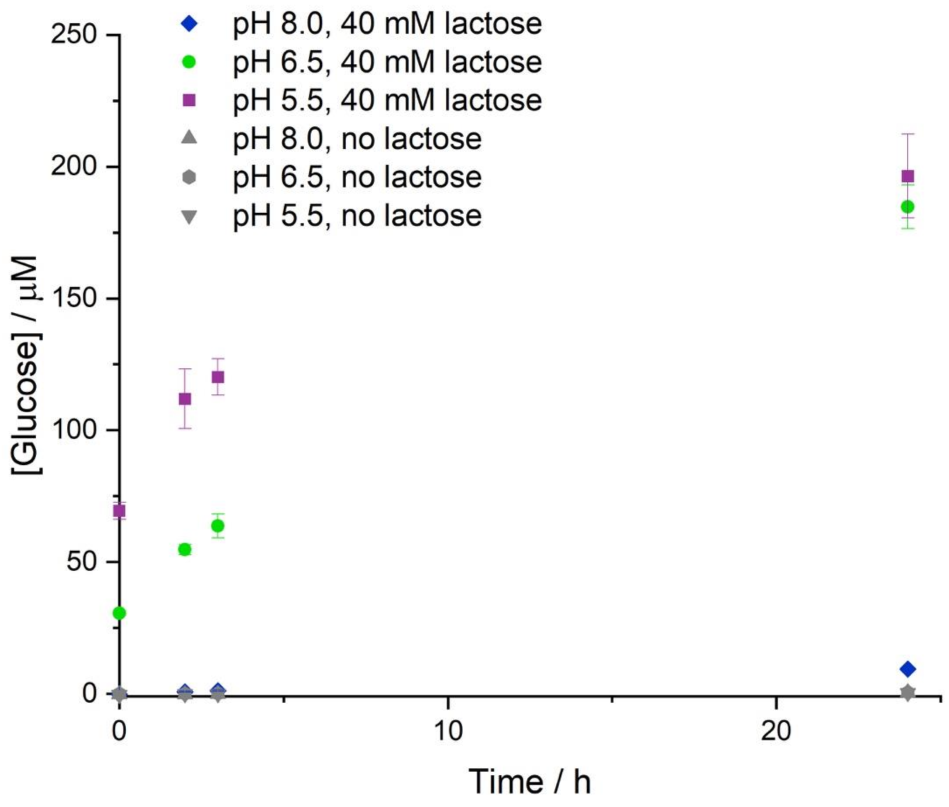

Supporting Figure 4. Generation of glucose by β-galactosidase activity at various pH values, with or without the lactose substrate. No glucose product was detected when the lactose input signal was not present (n = 3 for each pH condition). β-galactosidase was highly active at pH 5.5 (n = 3). Reduced activity was observed at pH 6.5 (n = 3), and very low activity at pH 8.0 (n = 3). Error bars represent standard deviation.

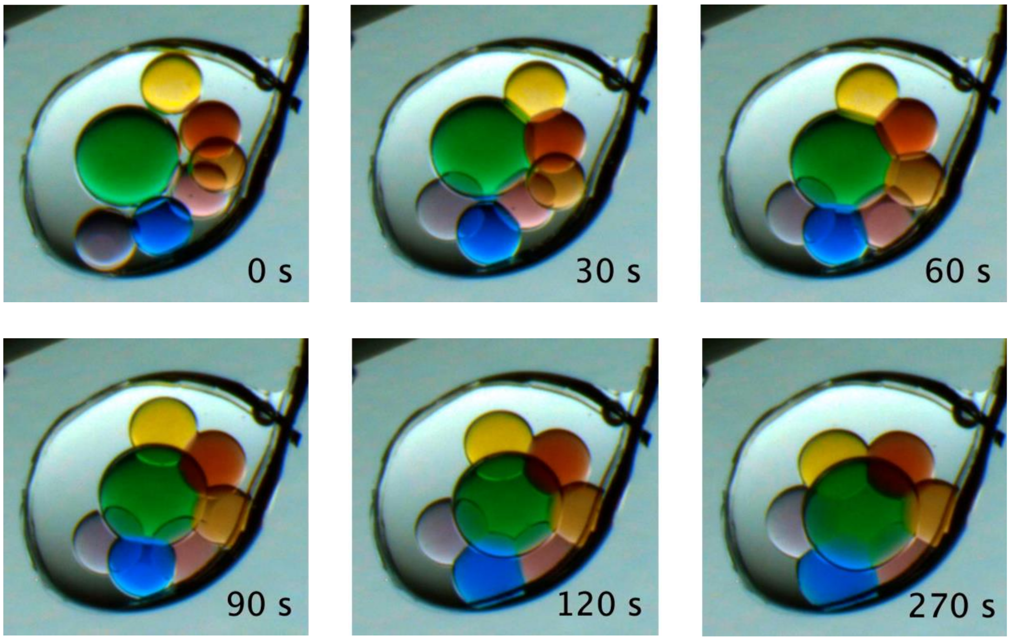

Supporting Figure 5. Generation of a multi-compartment processor mimic as seen in Supporting Video 3. A larger signal transmission compartment mimic in the middle (green dye) was connected to 6 processing compartment mimics (various colours). The final system self-assembled into the desired flower arrangement within 4.5 min. Wire diameter = 76 µm.

Supporting Notes

Considerations on Signal Transfer between Compartments

The transmission of chemical signals in our processors occurs by free diffusion of molecules through aqueous solutions and permeation through lipid membranes containing αHL. We can expect widely different times for equilibration of a signal molecule in a droplet interface bilayer (DIB) system depending on its chemical nature and the nature of the semipermeable membrane.

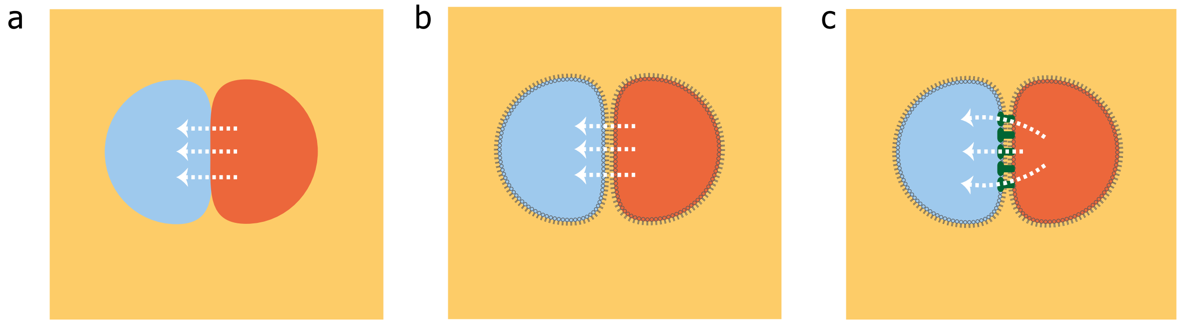

Supporting Figure 6. Equilibration of a signal molecule between compartments. **a.** Diffusion of molecules in the absence of a membrane. **b.** Diffusion of molecules by permeation through a lipid membrane. **c.** Diffusion of molecules through a lipid membrane permeabilized by αHL pores.

In the case of free diffusion in one dimension (**Supporting Figure 6a**), the equilibration time t may be approximated with the time lag equation:

$$t \approx \frac{\bar{x}^2}{6D} \qquad (1)$$

where $\bar{x}$ is the mean distance the solute diffuses and D is the diffusion coefficient of the solute in solution.[1]

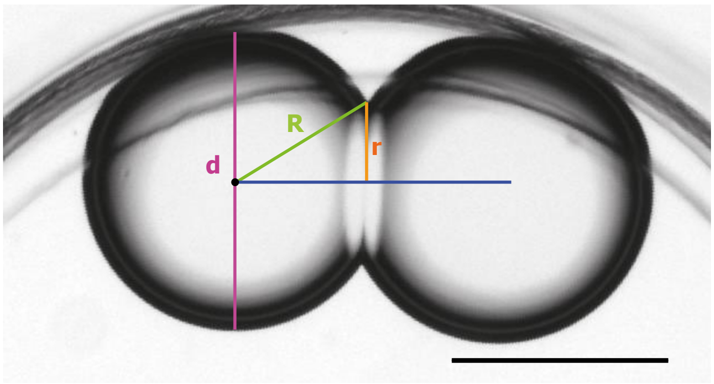

Supporting Figure 7. Overlays on an optical microscopy image of a DIB formed between two compartments, showing the compartment diameter, *d*; the compartment radius, *R*; and the DIB radius, *r*. Scale bar = 300 µm.

We first consider the hypothetical case of diffusion of glucose within two spherical compartments of diameter d = 410 µm. The diffusion coefficient of glucose in water[2] at 25 °C is $D \approx 650$ µm^2 s^{-1}. In this case, the mean distance $\bar{x}$ will be $\bar{x} \approx d$ = 410 µm (**Supporting Figure 7**). By applying Equation 1, we obtain $t \approx 43$ s. Therefore, for the equilibration of glucose by diffusion without a membrane, we expect an equilibration time of the order of tens of seconds.

We next consider the case where the two compartments are separated by a membrane (**Supporting Figure 6b**) with permeability P. In this scenario, the diffusive flux J of a solute through the membrane is given by:

$$J = -P\Delta C \qquad (2)$$

where ΔC is the difference between the concentrations of the solute across the membrane.[3] In our case shown in Supporting Figure 1, we have two compartments with 2-NBDG concentrations of C_1 = 1 mM and C_2 = 0 mM, respectively. In this case, $\Delta C = -1$ µmol cm^{-3}. Values of P vary widely,[4] *e.g.* for indole, a membrane-permeant molecule, $P_{indole} \approx 10^{-4}$ cm s^{-1}; for glucose, a membrane-impermeant molecule, $P_{glucose} \approx 10^{-7}$ cm s^{-1}.

To calculate the time required for equilibration, t, we need the DIB area A and the number of moles N that must diffuse through the membrane to achieve equilibration. For a compartment of d = 410 µm, the compartment radius will be R = 205 µm. Assuming a circular interface bilayer of radius $r = R / 2$ = 102.5 µm, the DIB area between the two compartments will be $A = \pi r^2 = 3.3 \times 10^{-4}$ cm^2. After equilibration, the two compartments will contain half the number of moles of solute initially in the compartment at concentration C_1. The number of moles to diffuse across the membrane is $N = C_1 V / 2$, where V represents the compartment volume. For a compartment of R = 205 µm, the volume is V = 36 nL = 3.6×10^{-5} cm^3. The number of moles to diffuse is therefore $N = 1.8 \times 10^{-5}$ µmol.

For a membrane-permeable small molecule (eg: indole), $P_{indole} \approx 10^{-4}$ cm s^{-1}, giving $J \approx 10^{-4}$ µmol cm^{-2} s^{-1}. The rate of diffusion is given by $J A \approx 3.3 \times 10^{-8}$ µmol s^{-1}. For $N = 1.8 \times 10^{-5}$ µmol, it takes $t \approx 550$ s (~9 min) for equilibration of the membrane-permeable solute. In reality, as ΔC becomes smaller throughout the process of diffusion, the equilibration time would be higher than this estimate.

For a membrane-impermeable small molecule (eg: glucose), $P_{glucose} \approx 10^{-7}$ cm s^{-1}, giving a flux $J \approx 10^{-7}$ µmol cm^{-2} s^{-1}. The rate of diffusion will then be $J A \approx 3.3 \times 10^{-11}$ µmol s^{-1}. For $N = 1.8 \times 10^{-5}$ µmol, it takes $t \approx 5.5 \times 10^5$ s (~6.3 days) for equilibration of the membrane-impermeable solute. Again, as ΔC becomes smaller throughout the process of diffusion, the real equilibration time would be higher than this estimate.

The permeabilization of the bilayer with αHL pores enables membrane-impermeant molecules to diffuse across the bilayer (**Supporting Figure 6c**). In Supporting Figure 1, we have shown equilibration within $t \approx 20$ minutes experimentally, demonstrating that the addition of the αHL pores permeabilized the membrane to a membrane-impermeant solute (2-NBDG).

Using equilibration time $t \approx 20$ minutes, we calculate the coefficient P of our αHL-permeabilized lipid membrane for 2-NBDG as:

$$P_{NBDG,aHL} = -\frac{J}{\Delta C} = -\frac{N}{A\,t\,\Delta C} \qquad (3)$$

Therefore, $P_{NBDG,aHL} \approx 4.6 \times 10^{-5}$ cm s^{-1}.

The calculation above underestimates $P_{NBDG,aHL}$ because the 20 min equilibration includes the time at the beginning of the process where the bilayer is still forming and the pores are still inserting (as shown in Supporting Figure 1).

Considerations on Signal Release into an External Bulk

As indicated throughout Supporting Note 1, the calculations performed only approximate the three scenarios in Supporting Figure 6. In reality, the rate of diffusion is not constant, but increases as the DIB forms and pores insert into the bilayer, and decreases with decreasing ΔC (as shown in Supporting Figure 1).

A more realistic description of diffusion can be made by using a differential equation that accounts for ΔC changing over time. For instance, we consider the two-compartment structure within an external aqueous environment in **Figure 3b**. Here, 2-NBDG diffuses out of its original compartment into the αHL-containing signal transmission compartment and then into the thousands of times larger bulk aqueous environment which serves as a sink. Hence, C_2, the concentration of 2-NBDG outside its original compartment stays negligible while the concentration inside the initial compartment, C_1, continues to decrease.

Based on Equation 3, J can be expressed as:

$$J(t) = \frac{1}{A}\frac{dN}{dt} \qquad (4)$$

The number of moles that need to diffuse is $N = V\,C_1$. Therefore, by substituting N in Equation 4, we obtain:

$$J(t) = \frac{V}{A}\frac{dC_1}{dt} \qquad (5)$$

The change in C_1 over time would therefore be:

$$\frac{dC_1}{dt} = \frac{J\,A}{V} = -\frac{P\,C_1(t)\,A}{\text{V}} \qquad (6)$$

Upon integration of Equation 6, we obtain:

$$\ln C_1 \Big|_{C_1(0)}^{C_1(t)} = -\frac{P\,A}{V}\Big|_0^t \qquad (7)$$

Equation 7 has the solution:

$$C_1(t) = C_1(0)\,e^{\frac{-PA}{V}t} \qquad (8)$$

The half time of this decay is:

$$t_{\frac{1}{2}} = \frac{V}{P\,A}\ln 2 \qquad (9)$$

Substituting the values for V, $P_{NBDG,aHL}$ and A from Supporting Note 1, we obtain:

$$t_{\frac{1}{2}} \approx 0.17 \times 10^4 \text{ s} \approx 28 \text{ min}$$

which is consistent with the results in Figure 3b that show complete release within minutes.

Effect of DIB Size on Signal Equilibration across the Membrane

In Supporting Note 1, we discussed equilibration of a molecule between compartments within an oil environment, assuming $r/R = 0.5$. The bilayer area is an important factor in determining the rate of diffusion. For R = 205 µm, we perform calculations with various DIB areas of $0.1 \le r/R \le 0.9$. Using $\Delta C = -1$ µmol cm^{-3}, N = 1.8 x 10^{-5} µmol and $P_{NBDG,aHL} \approx$ 4.6 x 10^{-5} cm s^{-1} from Supporting Note 1, we calculate the time of diffusion through the equation:

$$t \approx -\frac{N}{AP\Delta c} \qquad (4)$$

Supporting Table 1: Effect of bilayer area on equilibration of 2-NBDG across a DIB permeabilized by αHL, with constant compartment size.

r / R	r / µm	A / cm²	t / min
0.1	20.5	1.32E-05	500.0
0.2	41	5.28E-05	125.0
0.3	61.5	1.19E-04	55.6
0.4	82	2.11E-04	31.3
0.5	102.5	3.30E-04	20.0
0.6	123	4.75E-04	13.9
0.7	143.5	6.47E-04	10.2
0.8	164	8.45E-04	7.8
0.9	184.5	1.07E-03	6.2

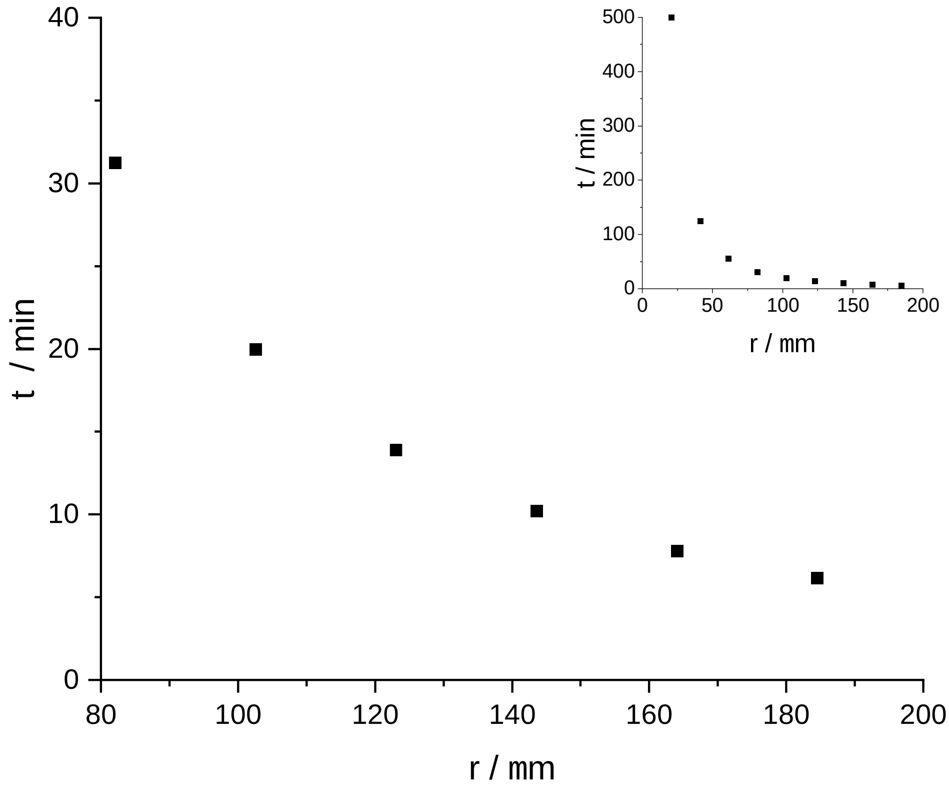

Supporting Figure 8: Effect of bilayer area, as indicated by bilayer radius *r*, on the expected time for equilibration *t* of 2-NBDG across a DIB permeabilized by αHL. Inset shows the equilibration times for a wider range of *r*.

Therefore, as the DIB area increases, the time taken for equilibration of solutes across a bilayer permeabilized by αHL decreases (**Supporting Table 1, Supporting Figure 8**).

Effect of Compartment Dimensions on Signal Equilibration across the Membrane

In Supporting Note 3, we calculated the expected equilibration times of 2-NBDG across a DIB permeabilized with αHL, joining compartments of R = 205 µm, as A is varied. We can also calculate the expected equilibration times with compartments of varying R. In this case, we assume the bilayer radius r is half the compartment radius, R. We calculate N as described in Supporting Note 1. Using Equation 1 and the ΔC and P values from Supporting Note 1, we obtain the expected equilibration times.

Supporting Table 2: Effect of compartment size on equilibration of 2-NBDG across a DIB permeabilized by αHL.

R / µm	r / µm	V / nL	A / cm^2	N	t / min
50	25	0.52	1.96E-05	2.62E-07	4.9
100	50	4.19	7.85E-05	2.09E-06	9.8
150	75	14.14	1.77E-04	7.07E-06	14.6
200	100	33.51	3.14E-04	1.68E-05	19.5
250	125	65.45	4.91E-04	3.27E-05	24.4
300	150	113.10	7.07E-04	5.65E-05	29.3
350	175	179.59	9.62E-04	8.98E-05	34.2
400	200	268.08	1.26E-03	1.34E-04	39.0

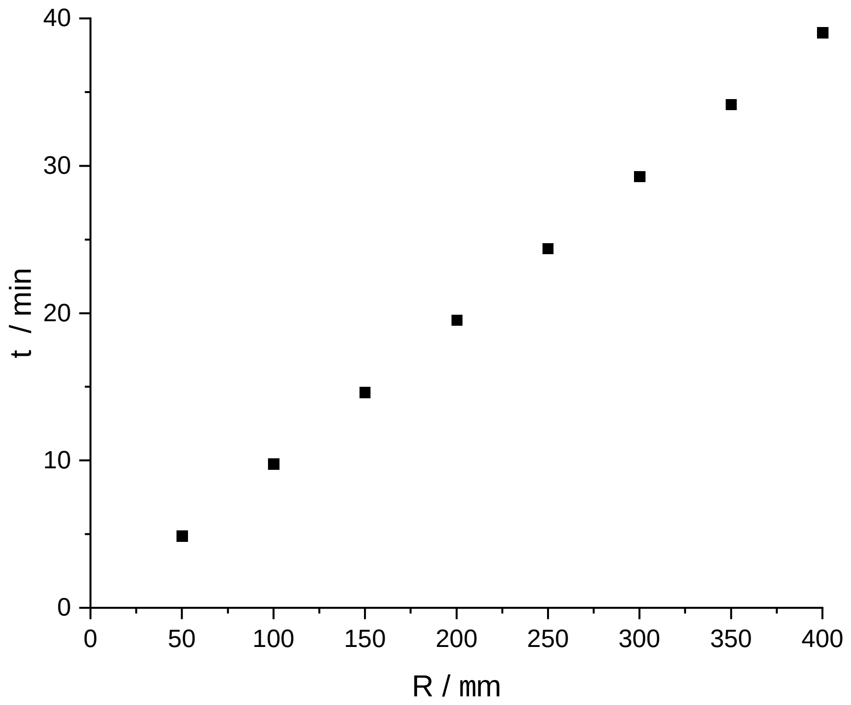

Supporting Figure 9: Effect of compartment size, as indicated by compartment radius R, on the time for equilibration t of 2-NBDG across a DIB permeabilized by αHL.

Depending on the size of the compartments ($50\ \mu m \leq R \leq 400\ \mu m$; $0.5\ nL \leq V \leq 268\ nL$), the equilibration time for 2-NBDG across a DIB permeabilized by αHL ranges from minutes to tens of minutes (**Supporting Table 2, Supporting Figure 9**).

As the compartment volume increases, the number of molecules increases by R^3, while the DIB area increases by R^2. Therefore, the expected time for equilibration increases by $R^3/R^2 = R$, which is also reflected in the linear trend observed in Supporting Figure 9.

Minimization of Background Fluorescence

To minimize the background fluorescence of Rhod-2 (K_d for $Ca^{2+} \approx 3.8\ \mu M$) due to trace metal ions in the aqueous solutions, we included 2 μM of a non-fluorescent chelator, 1,2-bis(*o*-aminophenoxy)ethane-*N,N,N',N'*-tetraacetic acid (BAPTA, K_d for $Ca^{2+} \approx 160$ nM) inside the sensing compartment and external aqueous environment. Unlike Rhod-2, BAPTA was not dextran-conjugated and could diffuse through the αHL pores. Over time, excess BAPTA from the external solution diffused into the sensing compartment and competed with the Rhod-2 to bind the trace ions, further reducing the fluorescence of Rhod-2.

Descriptions of Additional Supporting Files

Supporting Video 1. Composite bright field and epifluorescence microscopy time lapse of molecular diffusion from a signal release compartment containing 2-NBDG into a signal transmission compartment containing αHL, within an external lipid-oil environment, as seen in Supporting Figure 1. Each compartment was 410 μm in diameter. Molecular diffusion began immediately upon contact of the two compartments and proceeded simultaneously with bilayer formation. The fluorescence signal was mapped onto a spectrum colour map as indicated in Supporting Figure 1. Scale bars = 300 μm.

Supporting Video 2. Time lapse of molecular release from a two-compartment structure, which comprised a signal release compartment (250–300 μm in diameter) containing 2-NBDG and a signal transmission compartment (500–650 μm in diameter) containing αHL monomers, within an external aqueous environment, as seen in Figure 3b. The formation of a bilayer between the αHL compartment and the external aqueous environment was observed as a growing circle in the middle of the αHL compartment. Molecule release through the 2 bilayers was complete within 10 min.

Supporting Video 3. Time lapse of the formation of a multi-compartment processor mimic with one large signal transmission compartment and six small processing compartments, as seen in Supporting Figure 5. Within 270 s, the compartments self-assembled into a flower arrangement with each small compartment connected to the large compartment. Wire diameter = 76 μm.

References:

(1) Dudek, G.; Borys, P. A Simple Methodology to Estimate the Diffusion Coefficient in Pervaporation-Based Purification Experiments. *Polymers* **2019**, *11*, 343.

(2) Ribeiro, A. C. F.; Ortona, O.; Simões, S. M. N.; Santos, C. I. A. V.; Prazeres, P. M. R. A.; Valente, A. J. M.; Lobo, V. M. M.; Burrows, H. D. Binary Mutual Diffusion Coefficients of Aqueous Solutions of Sucrose, Lactose, Glucose, and Fructose in the Temperature Range from (298.15 to 328.15) K. *J. Chem. Eng. Data* **2006**, *51*, 1836–1840.

(3) Wayne, R. Plasma Membrane. In *Plant Cell Biology, 2nd ed.*; Academic Press, London **2019**, 17–53.

(4) Berg, J. M.; Tymoczko, J. L.; Stryer, L. *Biochemistry 5th ed.*; W. H. Freeman, New York, **2002**.
